# Outcomes of endoscopic and open resection of sinonasal malignancies: a systematic review and meta-analysis

**DOI:** 10.1016/j.bjorl.2021.06.004

**Published:** 2021-07-20

**Authors:** Sijie Jiang, Ruohao Fan, Hua Zhang, Weihong Jiang, Zhihai Xie

**Affiliations:** Xiangya Hospital of Central South University & Hunan Province Key Laboratory of Otolaryngology Critical Diseases, Department of Otolaryngology Head and Neck Surgery, Changsha, China

**Keywords:** Meta-analysis, Paranasal sinus neoplasms, Cancer of head and neck, Minimally invasive surgery, Surgical procedures

## Abstract

**Objective:**

To compare the efficacy of endoscopic and open resection of sinonasal malignancies.

**Methods:**

The search was performed using PubMed (1950–2020), Embase (1974–2020), the Cochrane library, and the website clinicaltrials.gov. The hazard ratio, HR, 95% confidence interval, CI, of the rates of overall survival and disease-free survival and the demographic characteristics of the included studies were extracted and analyzed. Pooled analysis was conducted with the studies’ individual patient data, using log-rank test, Kaplan–Meier survival, and Cox regression analysis.

**Results:**

Of 1939 articles retrieved, 23 articles were included. Overall, 1373 cases were incorporated into the final analysis, 653 (47.56%) of which underwent the surgery through an endoscopic approach, whereas 720 (52.44%) cases utilized the open approach. The overall survival was comparable between endoscopic and open resection (HR = 0.84 [95% CI: 0.65–1.07], *p* = 0.16; random effects analysis). Pooled analysis with Cox regression revealed signifcant differences in overall survival (HR = 0.568 [95%CI:0.380-0.849], *p* = 0.006) and disease-free survival (HR = 0.628 [95%CI:0.424-0.929], *p* = 0.02) between endoscopic and open approaches.

**Conclusion:**

The aggregated evidence suggests the survival outcome of endoscopic resection is comparable or greater than that of open resection of sinonasal malignancies.

## Highlights

• The overall survival rate of the endoscopic resection group was comparable with the open resection group.
• The disease-free survival rate of the endoscopic resection group was higher than the open resection group.
• The surgery approaches, the adjuvant therapy, the histopathology, and the T-stage have independent effects on the survival outcomes.

## Introduction

The concept of endoscopic endonasal surgery was first proposed in 1986 to deal with recurring rhinosinusitis.^1^ This approach had advantages such as better intraoperative vision, shorter recovery time, and potentially smaller postoperative facial scar or deformity.^2^ Sinonasal malignancies are known to be rare and carry a high risk of mortality. In 2000, Goffart applied endoscopic resection (ER) for the treatment of selected malignant sinonasal tumors, as he observed that there was little difference in the recurrence rate of benign lesions.^3^ Since then, endoscopy has been utilized in the treatment of sinonasal malignancies. However, it is yet to be discussed whether progressive margin resection, uncontrolled intraoperative hemorrhage, and the difficulty in skull base reconstruction, all of which occur in endoscopic resection, can increase the risk of mortality of the disease,^4^ especially advanced tumors. Meanwhile, with the development of high-definition endoscopy technology, the superiority achieved in implementation of endoscopy in malignancies cannot be neglected. Several meta-analyses have compared the outcome and efficacy of the endoscopic and open approaches in sinonasal malignancies indirectly, drawing a conclusion that the two approaches were comparable.^5^, ^6^ In a recent study, Lu arrived at a conclusion that the length of hospitalization was shorter in endoscopic endonasal surgery than in open resection (OR).^7^ In another meta-analysis, Hur demonstrated that endoscopic resection of sinonasal melanoma has better overall survival.^8^ However, due to the low incidence of sinonasal malignancies, the selection of the chosen surgical procedure in sinonasal malignancies is still to be discussed. The evidence-based implementation of endoscopic and open approaches remains to be explored due to the rarity and heterogeneity of sinonasal malignancies.

The purpose of our study was to conduct a meta-analysis of the current literature to compare the outcome of sinonasal malignancies via endoscopic and open approaches and to determine whether and when endoscopic approaches could achieve a comparable or better efficacy.

## Methods

### Search strategy

This systematic review and meta-analysis were conducted and reported based on the MOOSE (Meta-analysis of Observational Studies in Epidemiology) guidelines^9^ since all the trials involved were observational studies.

The search was performed using PubMed (1950–2020), Embase (1974–2020), the Cochrane library, and the website clinicaltrials.gov by two reviewers. The keywords used in the searching strategies included “sinonasal”, “malignancy”, “endoscopic”, and Medical Subject Headings (MeSH) terms, combined by Boolean operators. We retrieved literature from the reference lists of the obtained literature and contacted the authors by e-mail to include all the available studies.

### Inclusion and exclusion criteria

The following inclusion criteria were identified systematically in all the included studies: 1) The participants were diagnosed with sinonasal malignancies pathologically; 2) The participants received surgery with a curative intention and were allocated to the ER group (including endoscopic endonasal surgery and endoscopic-assisted surgery) or the OR group based on the surgical approach employed. Cases in each group were no less than 3 individuals; and 3) The hazard ratio (HR) and 95% confidence interval (CI) of the overall survival (OS) or disease-free survival (DFS) in each study were provided or could be calculated. Studies meeting the following criteria were excluded: 1) Tumor had not primarily originated from the nasal sinuses; and 2) Follow-up time was less than 12 months. Studies were included in a pooled-analysis when individual patient data were provided.

### Data extraction and statistical method

The HR and 95% CI of the rates of OS and DFS along with the demographic data including age, sex, diagnosis, stage of disease, statement of previous treatment, adjuvant therapy, and number of participants in each group were extracted from the included studies and aggregated by the reviewers independently. The HR and standard error (SE) were calculated using the methodology described by Tierney et al.^10^ when only the number of patients randomized into each arm of the trial, total number of events, and *p*-values of the log-rank test were provided. We also extracted data from Kaplan–Meier curves by tracing via the Engauge Digitizer software (version 12.1, free software downloaded from https://github.com/markummitchell/engauge-digitizer). Meta-analysis was conducted on the Review Manager software (version 5.3, free software downloaded from https://training.cochrane.org/online-learning/core-software-cochrane-reviews). Subgroup analyses based on previous treatment, pathology type, and comparability of studies were performed. When individual patient data were provided, a directive comparison was conducted using the SPSS software (version 23.0.0.0, IBM SPSS Statistics for Windows, Armonk, NY: IBM Corp). We categorized Kadish A/B and American Joint Committee on Cancer (AJCC) stages T1/T2 into “low stage”, and Kadish C/D and AJCC stages T3/T4 into “high stage”.^5^ The categorical variables were compared using a Chi-square test, whereas the continuous variables were compared using the Student’s *t*-test or Mann–Whitney *U*-test. Survival outcomes of both groups were compared using the Kaplan–Meier method, log-rank test, and the Cox regression analysis. A *p*-value of 0.05 or less was considered significant.

### Bias and quality assessment

Quality assessment for each study was evaluated using the Newcastle-Ottawa scale (NOS).^11^ The quality of evidence for each outcome was rated via Grading of Recommendations, Assessment, Development, and Evaluations (GRADE).^12^

## Results

**Figure 1:**
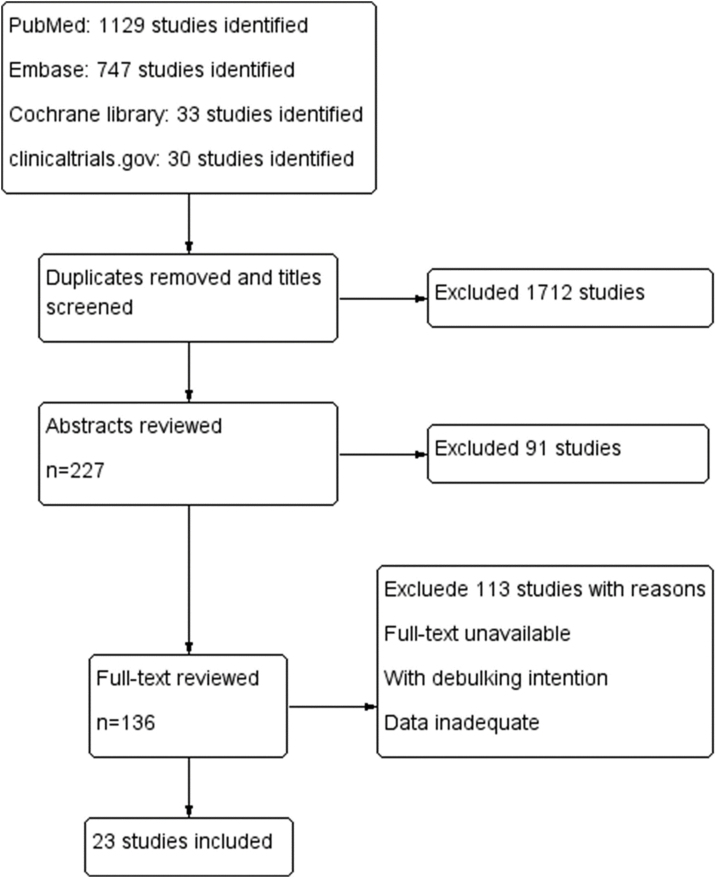
Flow diagram.

A total of 1939 articles were retrieved based on our search strategy. Of these, 227 articles were reserved after screening the title and removing the duplicates. After reviewing the abstracts, full-text analysis was carried out in 136 articles, according to the inclusion and exclusion criteria. Finally, 23 articles (Fig. 1) were included in the final meta-analysis, the characteristics of which are summarized in Table 1.^13^, ^14^, ^15^, ^16^, ^17^, ^18^, ^19^, ^20^, ^21^, ^22^, ^23^, ^24^, ^25^, ^26^, ^27^, ^28^, ^29^, ^30^, ^31^, ^32^, ^33^, ^34^, ^35^

**Table 1:** Studies included for meta-analysis.

Study	Year	Study type	Study period	Country	Nº of cases			Diagnosis (ER/OR)	Previous treatment	Adjuvant therapy		Mean age (y)	Nº of male	FU, mo	
					Total	ER	OR			ER	OR			ER	OR
Constantinidis, J.	2004	RC	1975–2000	Germany	23	11	12	ON	NA	8	9	51	NA	102.55	77.36
Orvidas, Laura J.	2005	RC	1980–2001	USA	23	3	20	AC	NA	2	20	71.33	17	36.33	73.87
Roth, T. N.	2010	RC	1992–2007	Switzerland	19	13	6	SNMM	NA	5	5	67.30	NA	46.46	28.50
Lund, V. J.	2012	RC	1963–2010	UK	109	31	78	SNMM	NA	NA	NA	NA	NA	NA	NA
Song, C. M.	2012	RC	1989–2008	South Korea	28	16	12	ON	NA	21	12	NA	NA	NA	NA
Guo, L.	2014	RC	1994–2011	China	23	8	15	CS (6/14), MYCS (1/0), MECS (1/1)	5	NA	NA	26.78	9	51.63	77.62
Saedi, B.	2014	RC	1999–2010	Iran	160	72	88	SCC (5/25), ACC (8/11), SNUC (4/9), ON (15/9), SNMM (21/8), ewing sarcoma (5/8), rhabdomyosarcoma (0/3), Sarcoma (0/3), transitional cell carcinoma (0/3), others (11/5)	0	38	65	47.60	112	22.00	20.00
Swegal, W.	2014	RC	1998–2012	USA	25	12	13	SNMM	6	11	11	65.50	14	32.40	46.80
Grosjean, R.	2015	RC	1998–2009	France	74	43	31	AC	0	NA	32	69.20	72	44.40	57.60
Ledderose, G. J.	2015	RC	2000–2010	Germany	22	12	10	SNMM	22	12	10	NA	NA	NA	NA
Won, T. B.	2015	RC	1994–2013	South Korea	133	70	63	SNMM	0	NA	NA	NA	NA	NA	NA
Cao, W.	2017	RC	1995–2014	China	33	15	18	SNMM	0	17	18	65.40	17	42.00	49.20
Hagemann, J.	2019	RC	1993–2015	Germany	225	123	102	SCC (51/52), AC (16/18), SNMM (17/11), ON (8/5), ACC (7/3), lymphomaa (6/0), sarcoma (7/4), SNUC (3/6), others (8/3)	NA	57	73	NA	135	54.40	45.40
Yin, G.	2019	RC	2004–2016	China	54	27	27	SNMM	0	20	13	57.07	28	28.37	25.33
Lai, Y.	2020	RC	2000–2016	China	92	57	35	SNMM	0	45	25	65.00	52	30.72	21.60
Lee, G.	2017	RC	1999–2015	South Korea	31	16	15	SNMM	0	12	13	NA	18	NA	NA
Batra, P. S.	2005	RC	1995–2003	USA	24	9	15	ON (0/8), SCC (2/5), AC (2/1), SNMM (2/0), SNUC (1/0), adenosquamous carcinoma (1/0), Sarcoma (1/1)	NA	12	18	NA	NA	NA	NA
Eloy, J. A.	2009	RC	1997–2006	USA	66	18	48	SCC (0/25), ON (10/4), ACC (3/8), AC (0/4), SNUC (1/2), SNMM (0/2), hemangiopericytoma (3/0), Sarcoma (0/2), small cell carcinoma (1/0), basal cell carcinoma (0/1)	NA	16	60	61.20	39	NA	NA
Mortuaire G.	2016	RC	2002–2013	France	43	20	23	AC	0	20	23	67.30	42	NA	NA
Bhayani, M. K.	2014	RC	1993–2009	USA	53	14	39	AC	NA	NA	NA	NA	NA	NA	NA
Vergez, S.	2012	RC	1999–2009	France	48	24	24	AC	NA	19	24	67.00	46	38.00	89.00
Huber, G. F.	2011	RC	1992–2007	Switzerland	18	12	6	AC	0	6	7	59.09	15	16.08	45.83
Huang, Y.	2018	RC	2001–2015	China	47	27	20	NA	NA	NA	NA	NA	NA	65.20	80.00

ACC, adenoid cystic carcinoma; AC, adenocarcinoma; CS, chondrosarcoma; DFS, disease-free survival; ER, endoscopic resection; FU, mean follow-up time; MECS, mesenchymal chondrosarcoma; MYCS, myxoid chondrosarcoma; NA, not available; NEC, neuroendocrine carcinoma; ON, esthesioneuroblastoma; OR, open resection; OS, overall survival; RC, retrospective cohort study; SCC, squamous cell carcinoma; SNMM, sinonasal melanoma; SNUC, sinonasal undifferentiated carcinoma.

### Meta-analysis

**Figure 2:**
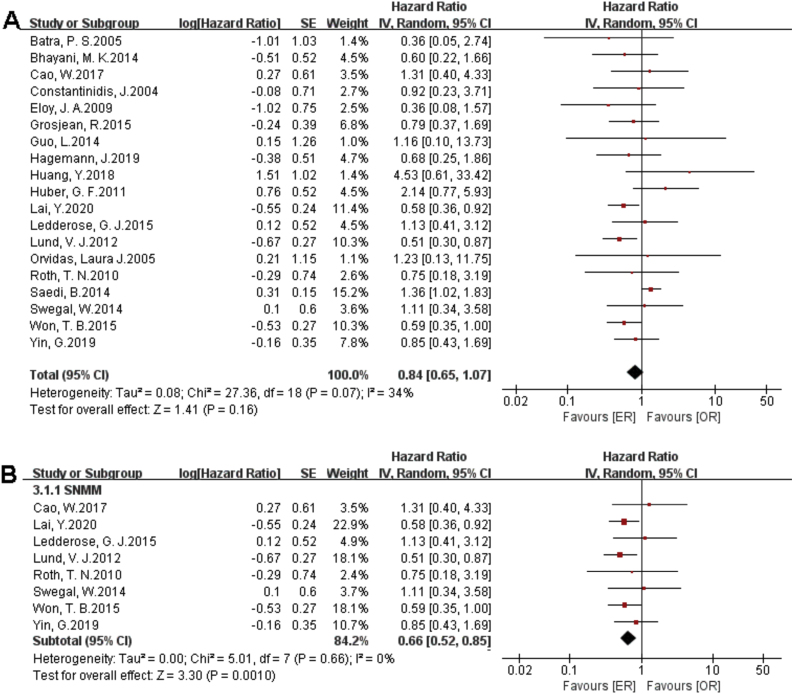
Comparison between endoscopic resection and open resection of sinonasal malignancies of overall survival and in (A) all studies and (B) sinonasal melanoma subgroups. CI, confidence interval; ER, endoscopic resection; OR, open resection.

**Figure 3:**
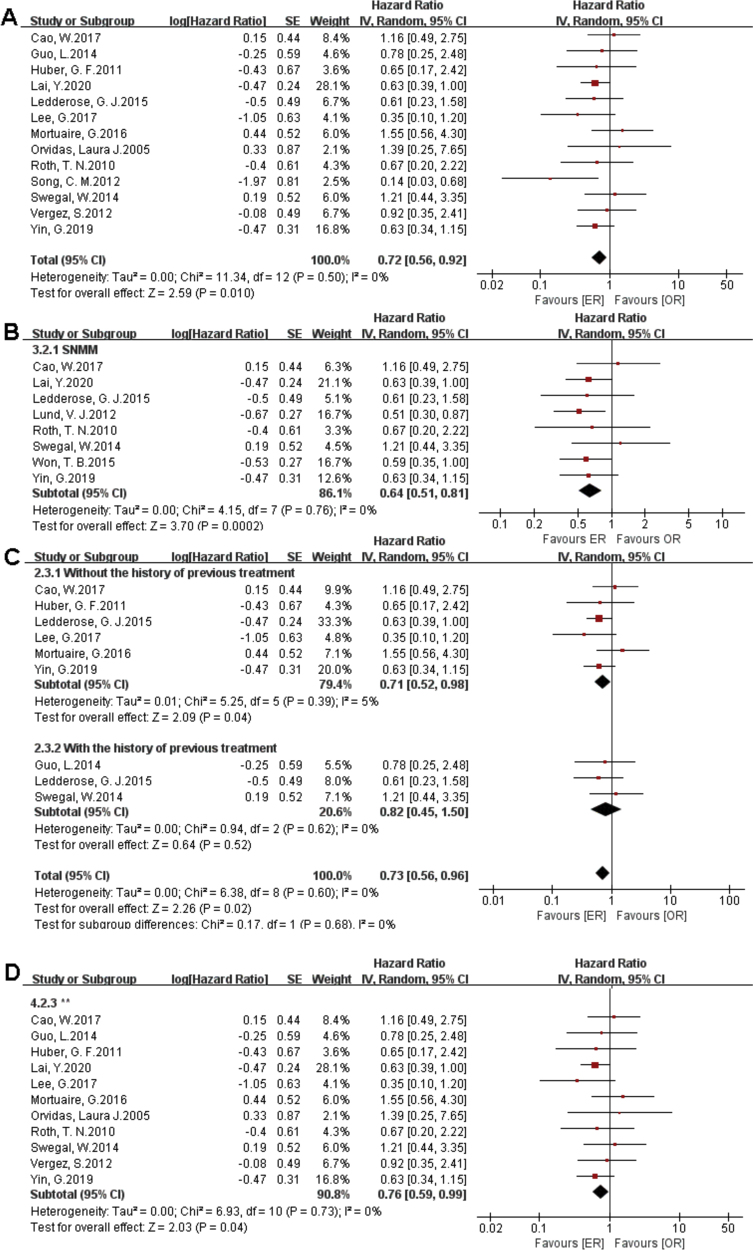
Comparison between endoscopic resection and open resection of sinonasal malignancies disease-free survival in (A) all studies, (B) sinonasal melanoma subgroups, (C) with or without previous treatment subgroups and (D) disease-free survival of comparability subgroups. CI, confidence interval; ER, endoscopic resection; OR, open resection; SNMM, sinonasal mucosal melanoma.

There were 1373 patients incorporated into our meta-analysis, of which 653 (47.56%) underwent surgery using the endoscopic approach, and 720 (52.44%) cases utilized open resection. Of the 23 articles included in the final meta-analysis, 19 studies (n = 1223 out of 1373) were included in the meta-analysis of OS. There was no significant difference in the OS between the endoscopic approach and the open approach (Fig. 2A), (HR = 0.84 [95% CI: 0.65–1.07], *p* = 0.16; random-effects analysis). Compared with the OR group, the OS rates in patients with sinonasal melanoma showed an advantage in the ER group (Fig. 2B), (HR = 0.66 [95% CI: 0.52–0.85], *p* = 0.001; random-effects analysis). Thirteen studies (n = 459 out of 1373) were included in the meta-analysis of DFS. The effect estimate suggested that the DFS of the ER group was higher than that of the OR group (Fig. 3A), (HR = 0.72 [95% CI: 0.56–0.92], *p* = 0.01; random-effects analysis). Compared with the OR group, the DFS rates in patients with sinonasal melanoma showed an advantage in the ER group (Fig. 3B) (HR = 0.64 [95% CI: 0.51–0.81], *p* = 0.0002; random-effects analysis). There was a significant difference in the DFS in cases without a previous treatment between the ER and OR groups (Fig. 3C), (HR = 0.71 [95% CI: 0.52–0.98], *p* = 0.04; random-effects analysis). The estimate effect of the HR of DFS favored the ER group in the subgroup with a higher comparability (Fig. 3D), (HR ≤ 0.76 [95% CI: 0.59–0.99], *p* = 0.04; random-effects analysis). There were no significant differences in the other subgroups (Supplementary Fig. 1).

### Pooled-analysis

**Figure 4:**
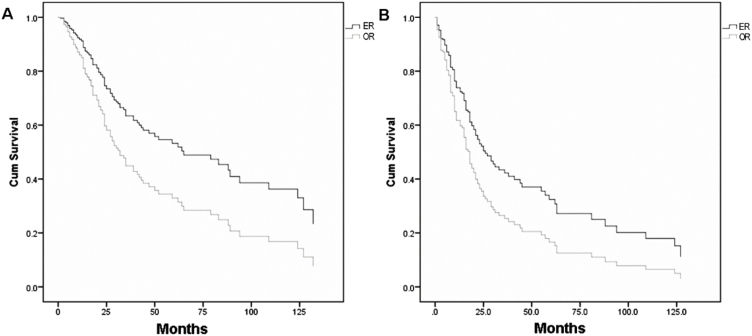
Survival curves of (A) overall survival between endoscopic resection and open resection (*p* = 0.006) and (B) disease-free survival between endoscopic and open resection (*p* = 0.020). ER, endoscopic resection; OR, open resection.

Table 2^13^, ^14^, ^15^, ^17^, ^18^, ^23^^,^^35^, ^36^, ^37^, ^38^ shows the individual patient data derived from 10 articles which met the inclusion criteria of pooled-analysis. A total of 248 cases were included in the pooled-analysis and went through a direct comparison. In pooled-analysis, OS of the ER and OR group was 31.7% and 21.1% (*p* < 0.05), respectively. Table 3 indicates significant differences of OS in age, pathological type, T-stage, and adjuvant therapy with univariate analysis and in T-stage, adjuvant therapy, and surgical approaches (*p* = 0.006) (Fig. 4A) with multivariate analysis. DFS of the ER and OR group was 19.9% and 15.5% (*p* < 0.05), respectively. Table 4 indicates significant differences in DFS in age, pathological type, T-stage, and adjuvant therapy with univariate analysis and in adjuvant therapy and surgical approaches (*p* = 0.020) (Fig. 4B) with multivariate analysis.

**Table 2:** Demographic data of pooled studies.

Variable	ER	OR	X^2^	*p*-Value
Age (mean ± SD)	58.09 ± 17.58	58.12 ± 19.22		0.99
Histopathology			16.25	0.002
Adenocarcinoma	15 (11.5%)	26 (22.0%)		
Chondrosarcoma	8 (6.2%)	13 (11.0%)		
Melanoma	70 (53.8%)	41 (34.7%)		
Esthesioneuroblastoma	30 (23.1%)	37 (31.4%)		
SNUC	7 (5.4%)	1 (0.8%)		
T stage			0.66	0.42
Low (T1–T2)	25 (21.0%)	14 (16.5%)		
High (T3–T4)	94 (79.0%)	71 (83.5%)		
Follow-up (median)	25.2	35.2		0.09
Adjuvant therapy			1.72	0.20
No adjuvant therapy	51 (41.8%)	32 (30.5%)		
Radiotherapy	31 (25.4%)	56 (53.3%)		
Chemotherapy	5 (4.1%)	2 (1.9%)		
Chemoradiotherapy	35 (28.7%)	15 (14.3%)		
Total	130 (52.4%)	118 (47.6%)		

ER, endoscopic resection; OR, open resection; SD, standard deviation; SNUC, sinonasal undifferentiated carcinoma.

**Table 3:** Cox proportional hazard analysis of overall survival.

Variable	Unadjusted			Adjusted		
	HR	95% CI	*p*	HR	95% CI	*p*
Group (ER vs. OR)	1.102	0.777–1.561	0.586	0.568	0.380–0.849	0.006
Age	1.034	1.023–1.046	<0.001	1.003	0.988–1.018	0. 710
Gender	1.014	0.83–1.239	0.889			
Pathology						
Esthesioneuroblastoma	Reference		<0.001			0.002
Melanoma	5.806	3.380–9.972	<0.001	3.407	1.665–6.973	0.001
SNUC	2.541	0.735–8.785	0.141	1.374	0.358–5.272	0.644
Adenocarcinoma	1.761	0.870–3.567	0.116	1.310	0.416–4.129	0.645
Chondrosarcoma	0.472	0.137–1.625	0.234			
Stage (high vs. low)	6.454	2.825–14.746	<0.001	2.716	1.030–7.164	0.043
Adjuvant therapy	2.032	1.428–2.891	<0.001	2.375	1.555–3.636	<0.001

CI, confidence interval; ER, endoscopic resection; HR, hazard ratio; OR, open resection; SNUC, sinonasal undifferentiated carcinoma.

**Table 4:** Cox proportional hazard analysis of disease-free survival.

Variable	Unadjusted			Adjusted		
	HR	95% CI	*p*	HR	95% CI	*p*
Group (ER vs. OR)	1.041	0.753–1.44	0.808	0.628	0.424–0.929	0.02
Age	1.026	1.016–1.037	<0.001	1.008	0.993–1.024	0.275
Gender	0.983	0.679–1.424	0.928			
Pathology						
Esthesioneuroblastoma	Reference		<0.001			0.004
Melanoma	6.224	3.433–11.282	<0.001	4.225	1.934–9.232	<0.001
Adenocarcinoma	2.533	1.271–5.049	0.008	3.117	1.228–7.911	0.017
Chondrosarcoma	2.109	0.953–4.665	0.066			
SNUC	2.029	0.573–7.181	0.273	2.494	0.652–9.541	0.182
Stage (High vs. Low)	5.207	2.53–10.72	<0.001	1.936	0.792–4.737	0.148
Adjuvant therapy	1.637	1.163–2.304	0.005	1.669	1.122–2.481	0.011

CI, confidence interval; ER, endoscopic resection; HR, hazard ratio; OR, open resection; SNUC, sinonasal undifferentiated carcinoma.

## Discussion

We conducted a meta-analysis of the available literature to compare the prognosis of sinonasal malignancies via endoscopic and open resection. Meanwhile a direct comparison was made between the groups from studies where the individual patient data was provided.

The meta-analysis indicated that the OS of the ER group was comparable with that of the OR group. This comparison of OS was, however, not stable. When we excluded Saedi’s study,^24^ the difference in the OS rates between the two groups turned into something meaningful (Supplementary Fig. 2), (HR = 0.72 [95% CI: 0.58–0.88], *p* = 0.002; random-effects analysis), which meant that the patients could benefit from ER in terms of OS rates. One explanation for the instability is that it arises from the relatively short follow-up time. The mean follow-up time of ER was 22 months, and that of OR was 20 months. However, the outcome of relapse requires a shorter follow-up time than death, which means OS needs longer follow-up time compared with DFS. In addition, the effect estimate suggested that DFS was higher when ER of sinonasal malignancies was performed.

The multivariate analysis of OS and DFS indicated a significant benefit of ER, which is different from univariate analysis. This variation may arise from the correlation between surgery approaches and the application of adjuvant therapy. There were 52.5% cases using adjuvant therapy in the endoscopic approach and 73.2% in the open approach (x^2^ = 7.559, *p* = 0.006). The multivariate analysis endorsed the application of adjuvant therapy as a protective factor. After eliminating the confounding factor through multivariate analysis, we found that surgery approaches have an independent effect on the survival outcomes. We are positive regarding the statistical result, considering the confidence interval of the effect estimate included appreciable benefit.

Rarity and heterogeneity of sinonasal malignancies contributed to the difficulty in the interpretation of survival results in the studies that reported different pathologies.^5^ Our multivariate analysis suggests that histopathology is an independent risk factor. A subgroup analysis was performed with studies where the pathological diagnosis was available. The effect estimate suggests that the outcome of the sinonasal melanoma in terms of OS and DFS is better for the endoscopic approach. In general, we believe that patients can benefit from ER. Since sinonasal melanoma is widely considered to be radioresistant, wide surgical excision is typically recommended as the primary mode of therapy.^39^, ^40^ However, endoscopic resection may be able to provide a better outcome by enabling excellent vision that offers precise excision and better local control. The effect estimate in adenocarcinoma subgroup suggests a comparable outcome in terms of OS and DFS.

There was a statistical correlation between T stage and survival.^41^ Previous studies have reported ER as an alternative to OR in low stage sinonasal malignancies.^5^ The tumor stage relates to tumor invasion extent, which is one of considerations when designing surgical approach. The effect of tumor stage in survival between endoscopic and open resection cannot be meta-analyzed as the sequence of the incompleteness of data, as well as the tumor invading site.

Adjuvant therapy plays a role in increasing the cure rate of sinonasal malignancies. Our multivariate analyses indicated that the adjuvant therapy was a protective factor for OS and DFS. Although the data in the literature provided were inadequate to conduct a subgroup analysis of the adjuvant therapy, the relationship between adoption of adjuvant therapy and selection of surgical approaches should not be underestimated. It is of much concern to develop a multidisciplinary therapy.

The advantages of endoscopic approach are technically clear. An endoscopic approach would be advocated for pathologies that surgical excision is recommended as primary therapy, based on the data summarized above. Meanwhile, endoscopic approach with or without auxiliary incision showed significant benefits for skull base involvement. But when an ocular enucleation or a total maxillectomy is required according to the extent of tumor, leading to inevitable facial deformity, an open surgical approach could benefit the patient. Lesions involving vital structures such as internal carotid artery are generally excised by the open approach according to the conventional viewpoint, but the endoscopic approach is an alternative due to the development of minimally invasive surgery technology and the improvement of surgical technique proficiency.

Of the 23 studies evaluated using NOS, 6 had 6 stars, 14 had 7 stars, and 3 had 8 stars (Table 5), whereas the maximum possible total score for a cohort study is 9 stars. The levels of evidence were accessed by the GRADEpro system. The certainties of effect estimate of OS and DFS were very low, on account of the imprecision and publication bias (Table 6). Moreover, the downgrading was on account of the following two aspects: 1) The confidence interval of the effect estimate contained an invalid value and included appreciable benefit^42^; and 2) The studies included in the analysis were observational studies. As a result, we were unable to ascertain whether the studies could represent all cases.^43^ However, due to the rarity of sinonasal malignancies, it would be difficult to plan a prospective randomized cohort study.

**Table 5:** Quality assessment of included studies by Newcastle-Ottawa assessment scale (NOS).

Study	Year	Nº of stars			
		Selection	Comparability	Outcome	Total
Constantinidis, J.	2004	3	1	2	6
Orvidas, Laura J.	2005	3	2	2	7
Roth, T. N.	2010	3	2	2	7
Lund, V. J.	2012	3	1	3	7
Song, C. M.	2012	3	1	2	6
Guo, L.	2014	3	2	3	8
Saedi, B.	2014	3	2	2	7
Swegal, W.	2014	3	2	2	7
Grosjean, R.	2015	3	1	2	6
Ledderose, G. J.	2015	3	1	2	6
Won, T. B.	2015	3	1	2	6
Cao, W.	2017	3	2	2	7
Hagemann, J.	2019	3	2	2	7
Yin, G.	2019	3	2	2	7
Lai, Y.	2020	3	2	2	7
Lee, G.	2017	3	2	2	7
Batra, P. S.	2005	3	2	2	7
Eloy, J. A.	2009	3	2	2	7
Mortuaire, G.	2016	3	2	2	7
Bhayani, M. K.	2014	3	1	2	6
Vergez, S.	2012	3	2	3	8
Huber, G. F.	2011	3	2	3	8
Huang, Y.	2018	3	2	2	7

**Table 6:** Outcomes assessment of included studies by GRADE.

Outcome	Certainty assessment							HR (95% CI)	Certainty	Importance
	Nº of studies	Study design	Risk of bias	Inconsistency	Indirectness	Imprecision	Other considerations			
OS	19	Observational studies	Not serious	Not serious	Not serious	Serious^a^	Publication bias strongly suspected^b^	0.84 (0.65–1.07)	⨁◯◯◯	Critical
									Very low	
DFS	13	Observational studies	Not serious	Not serious	Not serious	Serious^a^	Publication bias strongly suspected^b^	0.84 (0.61–1.14)	⨁◯◯◯	Critical
									Very low	

CI, confidence interval; DFS, disease-free survival; HR, hazard ratio; OS, overall survival.

a The OIS (optimal information size) criteria are met. But the confidence interval contains an invalid value and includes appreciable benefit.

b The cases included in analysis were observational studies. We could not make sure whether the studies could represent all cases.

There are some limitations of our study. First, the low quality of evidence is almost inevitable for observational studies, although the existence of some relevant factors could make it possible to improve the quality of the evidence, for example increasing the sample size to avoid imprecision. Second, the effects of adjuvant therapy, previous treatment, and histopathology were not analyzed adequately. Although subgroup analyses were planned to be conducted, the data reported by most of studies were deficient to perform such an analysis. Hence, further exploring the standardization of the reports would make sense.^5^, ^6^ At last, to the best of our knowledge, our study is the first one conducting a meta-analysis of the direct comparison between ER and OR groups. However, the effect estimate was not sufficiently stable. A longer follow-up time and more standard management are essential to improve the statistical power for further analysis.

## Conclusion

The evidence we collected suggests that the survival outcome of endoscopic resection in patients with sinonasal malignancies was comparable or better than that of open resection. The factors associated with tumor prognosis are histopathology, stage of tumor, and application of adjuvant therapy. Further research will be important to establish the guidelines for the selection of surgical approach and promote the comprehensive treatment of sinonasal malignancies.

## Conflicts of interest

The authors declare no conflicts of interest.
