# Supplementary material for: Outcomes of endoscopic and open resection of sinonasal malignancies: a systematic review and meta-analysis
Source: Braz J Otorhinolaryngol. 2021 Jul 20;88(Suppl 5):S19–31. doi: 10.1016/j.bjorl.2021.06.004 (PMC9800954; doi:10.1016/j.bjorl.2021.06.004)

**BJORL-D-21-00231 – Supplementary Material**

**Supplement Figure 1** Comparison between endoscopic resection and open resection of sinonasal malignancies in (A) overall survival of with or without previous treatment subgroups and (B) overall survival of comparability of 1 star or 2 stars. CI, Confidence Interval; ER, Endoscopic Resection; OR, Open Resection; SNMM, Sinonasal Mucosal Melanoma; AC, Adenocarcinoma.


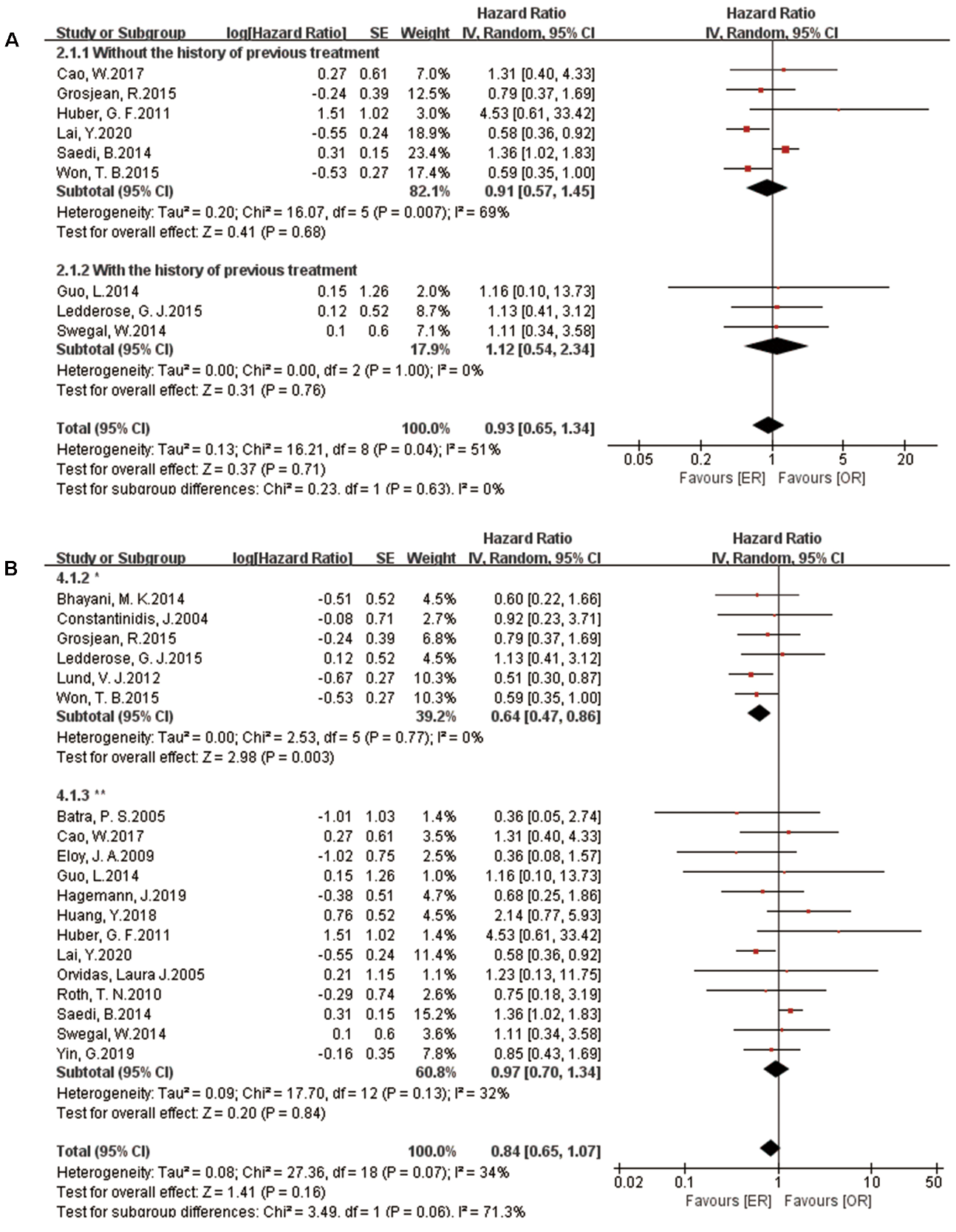


**Supplement Figure 2** Comparison between endoscopic resection and open resection of sinonasal malignancies in overall survival with Saedi’s study excluded. ER, Endoscopic Resection; OR, Open Resection.


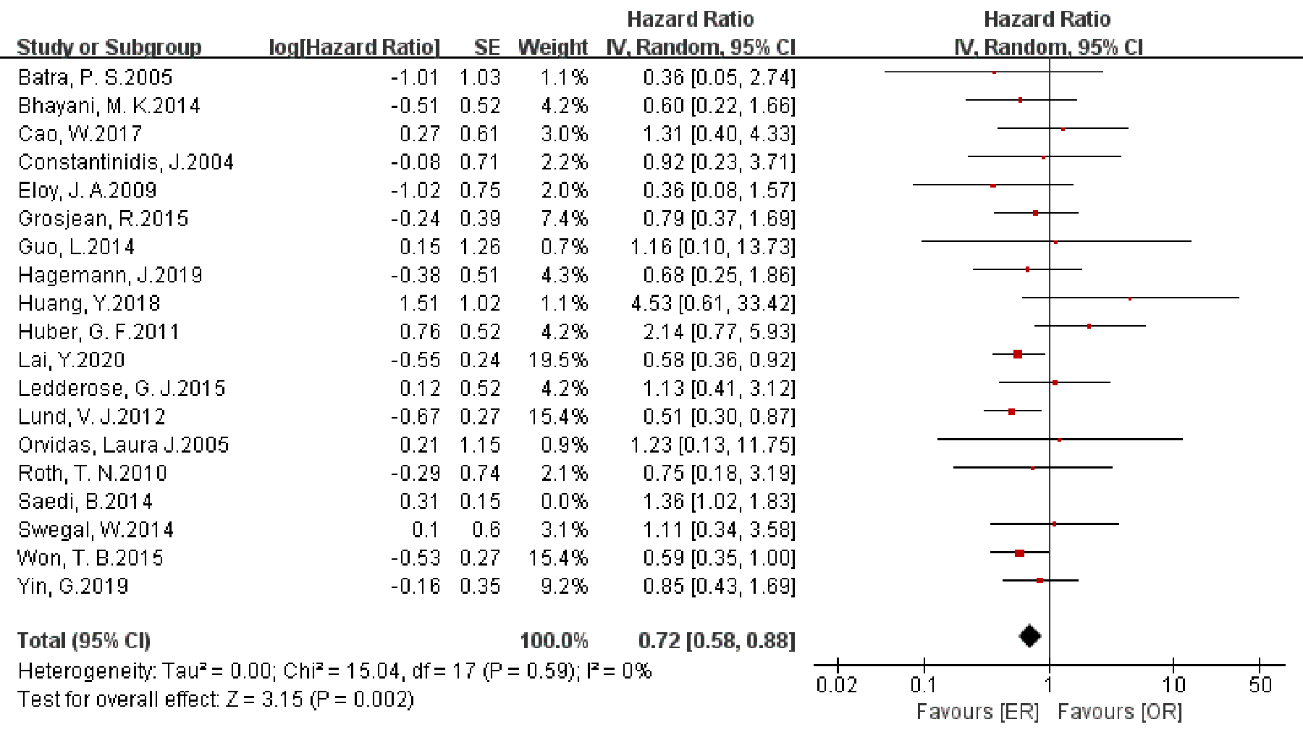

Supplement: Supplementary file 1 [file mmc1.docx]
